# Pseudohyphal Growth of the Emerging Pathogen Candida auris Is Triggered by Genotoxic Stress through the S Phase Checkpoint

**DOI:** 10.1128/mSphere.00151-20

**Published:** 2020-03-11

**Authors:** Gustavo Bravo Ruiz, Zoe K. Ross, Neil A. R. Gow, Alexander Lorenz

**Affiliations:** a Institute of Medical Sciences, University of Aberdeen, Aberdeen, United Kingdom; b MRC Centre for Medical Mycology, Department of Biosciences, University of Exeter, Exeter, United Kingdom; University of Georgia

**Keywords:** *Candida auris*, filamentous growth, S phase checkpoint, Rad51, Rad9, Mrc1

## Abstract

The morphogenetic switching between yeast cells and filaments (true hyphae and pseudohyphae) is a key cellular feature required for full virulence in many polymorphic fungal pathogens, such as Candida albicans. In the recently emerged yeast pathogen Candida auris, occasional elongation of cells has been reported. However, environmental conditions and genetic triggers for filament formation have remained elusive. Here, we report that induction of DNA damage and perturbation of replication forks by treatment with genotoxins, such as hydroxyurea, methyl methanesulfonate, and the clinically relevant fungistatic 5-fluorocytosine, cause filamentation in C. auris. The filaments formed were characteristic of pseudohyphae and not parallel-sided true hyphae. Pseudohyphal growth is apparently signaled through the S phase checkpoint and, interestingly, is Tup1 independent in C. auris. Intriguingly, the morphogenetic switching capability is strain specific in C. auris, highlighting the heterogenous nature of the species as a whole.

**IMPORTANCE** Candida auris is a newly emerged fungal pathogen of humans. This species was first reported in 2009 when it was identified in an ear infection of a patient in Japan. However, despite intense interest in this organism as an often multidrug-resistant fungus, there is little knowledge about its cellular biology. During infection of human patients, fungi are able to change cell shape from ellipsoidal yeast cells to elongated filaments to adapt to various conditions within the host organism. There are different types of filaments, which are triggered by reactions to different cues. Candida auris fails to form filaments when exposed to triggers that stimulate yeast filament morphogenesis in other fungi. Here, we show that it does form filaments when its DNA is damaged. These conditions might arise when Candida auris cells interact with host immune cells or during growth in certain host tissues (kidney or bladder) or during treatment with antifungal drugs.

## Abstract (precis)

Candida auris is a newly emerged fungal pathogen of humans. This species was first reported in 2009 when it was identified in an ear infection of a patient in Japan. However, despite intense interest in this organism as an often multidrug-resistant fungus, there is little knowledge about its cellular biology. During infection of human patients, fungi are able to change cell shape from ellipsoidal yeast cells to elongated filaments to adapt to various conditions within the host organism. There are different types of filaments, which are triggered by reactions to different cues. Candida auris fails to form filaments when exposed to triggers that stimulate yeast filament morphogenesis in other fungi. Here, we show that it does form filaments when its DNA is damaged. These conditions might arise when Candida auris cells interact with host immune cells or during growth in certain host tissues (kidney or bladder) or during treatment with antifungal drugs.

## INTRODUCTION

The emergence of novel multidrug-resistant pathogens poses a recurrent global threat to health care settings. This is the case for the fungus Candida auris discovered as a new human pathogen only 10 years ago (1), albeit a retrospective review of strain collections dated the first case back to 1996 (2). Since its first identification, C. auris has been found across all continents, causing clonal outbreaks in hospital settings (3). It shows mortality rates in systemic disease close to 50% (4), is one of the most drug-resistant yeast pathogens (5), and has been described as a skin colonizer able to undergo nosocomial spread (6), thus becoming a major concern for medical mycology.

Due to the recent emergence of this pathogen, we are largely ignorant about its general biological traits. This lack of fundamental understanding about the origin (7, 8) and the life cycle of C. auris impedes our capacity to explain its sudden emergence, rapid global spread, and unique phenotypic characteristics. An example is the lack of information about its ability to undergo morphogenetic switches as described for other fungi. In fungi, a morphogenetic switch enables cells to change from growing as unicellular yeasts to pseudohyphae or true hyphae and can be triggered by a multitude of environmental factors, such as nutrient limitation, temperature, and pH changes (reviewed in references [Bibr B9][Bibr B10][Bibr B12]). Filamentous growth allows the exploration of new environments and is considered a virulence trait in pathogenic fungi (reviewed in references 13 and 14). However, most cues causing filamentation in the best-studied and only distantly related pathogen Candida albicans do not induce filamentous growth in C. auris (15).

In contrast to growth of true hyphae, pseudohyphal growth has been associated with a delay in cell cycle progression and the subsequent extension of the apical growth period (16). Indeed, it has been demonstrated that drugs causing genotoxic stress, such as hydroxyurea (HU) or methyl methanesulfonate (MMS), trigger S phase arrest via a cell cycle checkpoint (reviewed in reference 17); this results in pseudohyphal growth in C. albicans and Saccharomyces cerevisiae (18, 19). The S phase checkpoint is a surveillance system, which responds to DNA damage or DNA replication fork arrest, and involves the sensor kinases Mec1 and Tel1, the mediator proteins Rad9 and Mrc1, and an effector kinase, Rad53. Rad9 acts as the main mediator for the DNA damage response, whereas Mrc1 functions as a DNA replication arrest responder (17). Once activated, the S phase checkpoint modulates multiple biological processes, including the repression of late-firing replication origins, cell cycle progression, the production of deoxynucleotide triphosphates (dNTPs), the transcription of DNA damage response genes, and inhibition of homologous recombination. Accordingly, activation of Rad53 triggers pseudohyphal growth since in S. cerevisiae or C. albicans mutants deficient in this kinase showed a drastic reduction of filamentation under genotoxic stress conditions (19, 20).

Here, we demonstrate that many, but not all, clinical isolates of C. auris are capable of pseudohyphal growth when treated with genotoxins such as HU, MMS, or the clinically relevant fungistatic 5-fluorocytosine (5-FC). Deletion mutants of genes involved in the S phase checkpoint, *RAD9* and *MRC1*, or homologous recombination, *RAD51* and *RAD57*, allowed us to probe whether a functional S phase checkpoint is required for filamentous growth in C. auris. Our work provides the first insight into how genome stability maintenance supports cell growth and proliferation and what triggers morphogenetic switching in the newly emerged fungal pathogen of humans, C. auris.

## RESULTS

### C. auris produces pseudohyphae under genotoxic stress.

**FIG 1:**
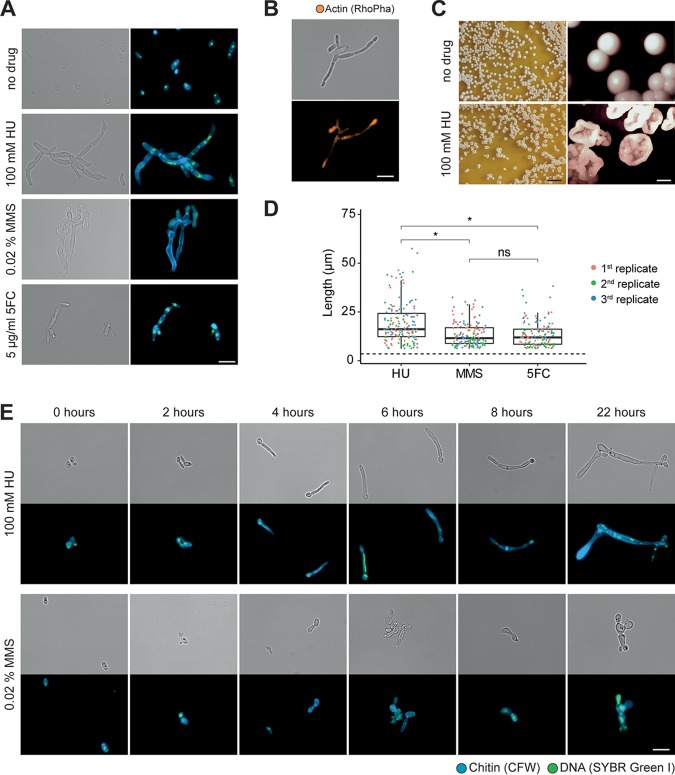
Filamentation of Candida auris (UACa11) in the presence of genotoxic drugs. (A) Microscopy images of C. auris filamentation after growing strain UACa11 cells on YPD plates with or without the addition of the indicated drug after 3 days at 30°C. A bright-field image is shown on the left, and a merged fluorescent image (chitin stained by calcofluor white [blue] and DNA stained by SYBR green I [green]) is shown on the right. (B) Cells grown on YPD plates containing 100 mM HU for 3 days at 30°C stained for actin using rhodamine-phalloidin (RhoPha) (bottom image). The bright-field image is on top. (C) Details of colonies from strain UACA11 grown on YPD plates in the absence and presence of 100 mM hydroxyurea (HU) for 6 days at 30°C. Scale bars, 10 mm (left) and 1 mm (right). (D) Lengths of filaments of wild-type (UACa11) cells grown in YPD medium containing 100 mM HU, 0.02% MMS, or 5 μg/ml 5-FC for 18 to 20 h at 30°C (*n* = 50 for each replicate). Only cells longer than 6 μm were considered filaments. The dotted line indicates the average length of 300 yeast cells (wild-type UACa11 grown in YPD medium for 18 to 20 h at 30°C). *, *P < *0.05 (by Wilcoxon rank sum test); ns, not significant. (E) Microscopy images of representative filaments of C. auris UACa11 formed in liquid culture. Cells were stained as described for panel A. After arrest in G_1_, cultures were grown for 165 min in YPD medium before the indicated drugs were added (time point 0 h). Bright-field images are shown in the top rows, and fluorescent images are shown at bottom. Scale bar, 10 μm.

Because the ability to switch between unicellular and filamentous forms plays a role in pathogenesis in some fungi (reviewed in references 11 and 12), we were interested in whether C. auris has the capability to form filaments as well. Several conditions which induce hyphal growth in C. albicans, such as incubation at 37°C and in Lee’s medium at pH 3.5 or pH 6.5, as well as medium containing serum, isoamyl alcohol, or Bleocin, were tested, but none of these triggered filamentous growth in the South Asian (clade I) C. auris strain UACa11 (data not shown) (15). Likewise, growth at 25°C did not produce filaments as described previously for a C. auris clinical isolate (21). However, using strain UACa11, we observed filamentous growth in the presence of sublethal concentrations of genotoxic drugs affecting DNA replication progression or inducing DNA damage (HU, MMS, and 5-FC) (Fig. 1) (see also Fig. S1 in the supplemental material; all supplemental figures and tables can be found at https://doi.org/10.6084/m9.figshare.11378550). HU inhibits the activity of ribonucleotide reductase (22) and thus induces a depletion of the dNTP pools (23). MMS creates bulky adducts by alkylating DNA that interferes with fork progression (24). 5-FC is converted into fluorouracil in the cell and perturbs RNA and DNA biosynthesis (25). The filaments observed in C. auris show characteristics attributed to pseudohyphae (9, 11). In contrast to true hyphae, filaments in C. auris are wider than the diameter of a yeast cell and do not present parallel sides, septa between neighboring cells show visible indentations, and actin patches are not accumulating at the growing tip (Fig. 1). Moreover, nuclear divisions seem to occur at mother-daughter junctions, but this phenotype is often complicated by nuclear division defects due to the genotoxin treatments (Fig. 1).

Within 6 to 8 h of growth in yeast extract-peptone-glucose (YPD) medium containing 100 mM HU, daughter cells started to show hyperpolarized growth. This results in almost 100% filamentation after overnight culture (Fig. 1E; see Fig. S1A at the URL mentioned above), similar to C. albicans (18, 20). In this early filamentation phase, generally one or two nuclei (SYBR green-stained DNA signals) were observed within the same cell, and occasionally very large, high-intensity or stretched out DNA signals could be seen (Fig. 1E). After 22 h constrictions at septa became evident throughout the filaments, and new buds emerged from seemingly random locations along the filament; these cultures contained almost no separated (yeast) cells, indicating a defect in cell separation as expected for pseudohyphal growth (Fig. 1E; Fig. S1A). Cells were often multi- or anucleate, suggestive of karyokinesis defects. A similar phenotype was generated by the addition of 0.02% MMS (Fig. 1E) although the formation of filaments occurred more slowly, and the resulting pseudohyphae tended to be shorter and more irregular than those with HU treatment (Fig. 1D). Moreover, after 22 h in MMS, round and elongated yeast cells were still present in the cultures. Importantly, the number and length of filaments decreased over time when cells were grown on plates containing genotoxins (Fig. S2). This means either that long filaments might not be viable and that surviving yeast cells start growing after adapting their checkpoint or that filaments bud off yeast cells, which then form a new population. It should also be kept in mind that drugs could be degraded over time and that after several days the environment on solid medium containing genotoxic drugs could be less challenging. Filaments can also be observed without stress, as previously reported (26), but these are rare and could be explained by sporadic DNA damage (see Fig. S1A at the URL mentioned above). Strikingly, sporadically giant round cells were also observed in the presence of genotoxic drugs (Fig. S1A and S2). In accordance with the cellular phenotype, the colony morphology after 5 to 6 days on solid medium is rougher when HU is present, lacking the typical smooth-colony appearance (Fig. 1C). This was not true in the presence of MMS or 5-FC, either because these filaments were shorter or less abundant or because they were more short-lived than the ones treated with HU (Fig. S2).

### C. auris lacks key genes associated with hyphal growth.

Filamentation is a complex mechanism, usually triggered by environmental conditions, involving hundreds of genes in S. cerevisiae and C. albicans (27–29). Among them is a group of well-studied key regulators known as hypha-specific genes (HSGs). Previously, Muñoz and coworkers reported that some HSGs essential for true hyphal growth, such as *ECE1* or *HWP1*, are absent in the C. auris genome (30). We further explored the presence of key genes in filamentation in the C. auris genome (see Table S1 at https://doi.org/10.6084/m9.figshare.11378550). Besides *ECE1* and *HWP1*, *FLO11*, *EED1*, and *HWP2* were also absent in C. auris. In S. cerevisiae Flo11 is a key factor for pseudohyphal growth in response to nutrient limitation (31, 32). Intriguingly, in C. albicans, which also lacks Flo11, Hwp2 seems to cover this function (33). *UME6*, which codes for a Zn(II)2Cys6 transcription factor involved in filamentation regulation, is present in *Candida* species capable of forming true hyphae (C. albicans, C. tropicalis, and C. dubliniensis) and species unable to form true hyphae (C. parapsilosis, C. orthopsilosis, and C. lusitaniae). Importantly, in the latter group of species only the C-terminal Zn(II)2Cys6 domain is well conserved; this is also true for C. auris (see Fig. S3 at the URL mentioned above).

**FIG 2:**
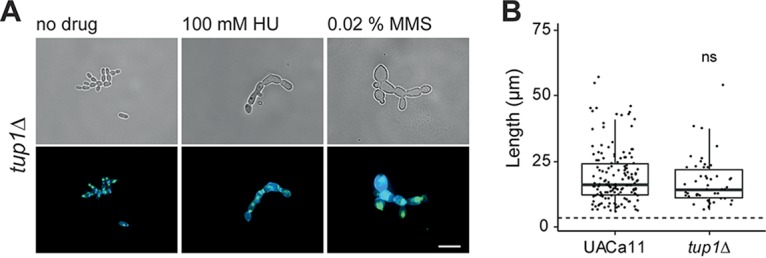
Microscopic analysis of filament formation in Candida auris *tup1* mutant (UACa11 background). (A) Representative microscopic images of C. auris filaments after growing *tup1*Δ cells in YPD broth with or without the addition of the indicated drug for 18 to 20 h at 30°C (top, bright-field images; bottom, merged fluorescent images showing chitin stained by calcofluor white [blue] and DNA stained by SYBR green I [green]). Scale bar, 10 μm. (B) Length of filaments observed after growth of wild-type (UACa11) (*n* = 150) and *tup1*Δ (*n* = 50) cells in YPD broth containing 100 mM HU for 18 to 20 h at 30°C. Only cells longer than 6 μm were considered filaments. The dotted line indicates the average length of 300 yeast cells (wild-type UACa11 grown in YPD for 18 to 20 h at 30°C). ns, not significant (Wilcoxon rank sum test).

Tup1 is a transcriptional repressor for filamentous growth in C. albicans acting on several HSGs, and *tup1*Δ strains grow constitutively as true hyphae (34, 35). In a C. auris *tup1*Δ strain, no filaments were observed when cells were grown without stress (Fig. 2). However, strings of yeast cells were frequently observed, indicating that cells cannot separate properly. Under genotoxic stress, the *tup1*Δ mutant formed pseudohyphae similar to those of the parental strain. Altogether, this suggests that filamentation in C. auris is likely regulated differently than that in C. albicans.

### C. auris has a functional S phase checkpoint.

Genotoxic drugs induce replication fork perturbations and/or DNA damage, leading to cell cycle arrest by triggering the S phase checkpoint. Genes encoding S phase checkpoint factors, *MEC1* (GenBank accession number XP_028890424), *RAD9* (XP_028889586), *MRC1* (XP_028891779), and *RAD53* (XP_028891118), and homologous recombination proteins, *RAD51* (GenBank accession number XP_028892133) and *RAD57* (KND99929), were found in the C. auris genome (see Fig. S4 and S5 and Table S1 at the URL mentioned above). Null mutants of *RAD9*, *MRC1*, *RAD51*, and *RAD57* were obtained by deleting the open reading frames (ORFs) in the C. auris UACa11 strain (Fig. S6). Unfortunately, three attempts to generate *mec1*Δ or *rad53*Δ null mutants with >300 transformants each were unsuccessful, possibly because these genes are essential in C. auris. The *rad51*Δ and *rad57*Δ strains showed very similar phenotypes (see Fig. S7 at the URL mentioned above); for detailed analysis we focused on the *rad51*Δ strain.

**FIG 3:**
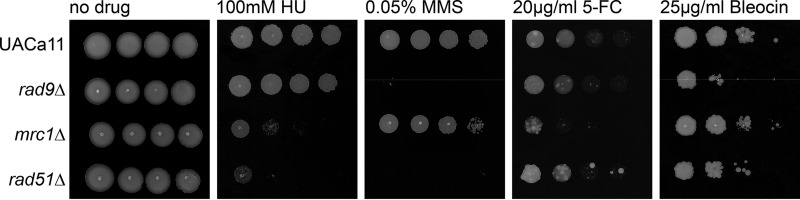
Candida auris *rad9*, *mrc1*, and *rad51* mutants show sensitivity to genotoxic drugs. Growth analysis by spot assays of the wild type (UACa11) and the indicated deletion mutants in the presence and absence of genotoxic drugs. Ten-fold serial dilutions of C. auris cells were grown on YPD plates containing the indicated drug for 3 days at 30°C.

The mutant phenotypes were characterized using sublethal concentrations of various genotoxic drugs (Fig. 3). None of the mutants showed a conspicuous growth defect in the absence of genotoxins (Fig. 3). The *rad9*Δ strain showed sensitivity only to MMS and Bleocin, indicating a role in responding to double-stranded DNA breaks (Fig. 3). In contrast, the *mrc1*Δ strain displayed sensitivity to drugs inducing replication-associated damage (HU, MMS, and 5-FC) but grew like the parental control strain on Bleocin (Fig. 3). Growth of *rad51*Δ was severely affected in the presence of HU and MMS but only moderately so in the presence of Bleocin, and growth was almost indistinguishable from that of the wild type on 5-FC (Fig. 3). These mutant C. auris phenotypes are similar to the ones observed in the corresponding S. cerevisiae and C. albicans mutants (20, 36–39). However, Mrc1 seems to play a lesser role in C. auris since in the other two yeasts *mrc1*Δ strains were more sensitive to MMS, and in C. albicans this mutant showed a growth defect even when genotoxic challenges were absent (20, 36–39). Taken together, these results suggest that the S phase checkpoint is conserved in C. auris, albeit with a somewhat reduced importance of Mrc1.

**FIG 4:**
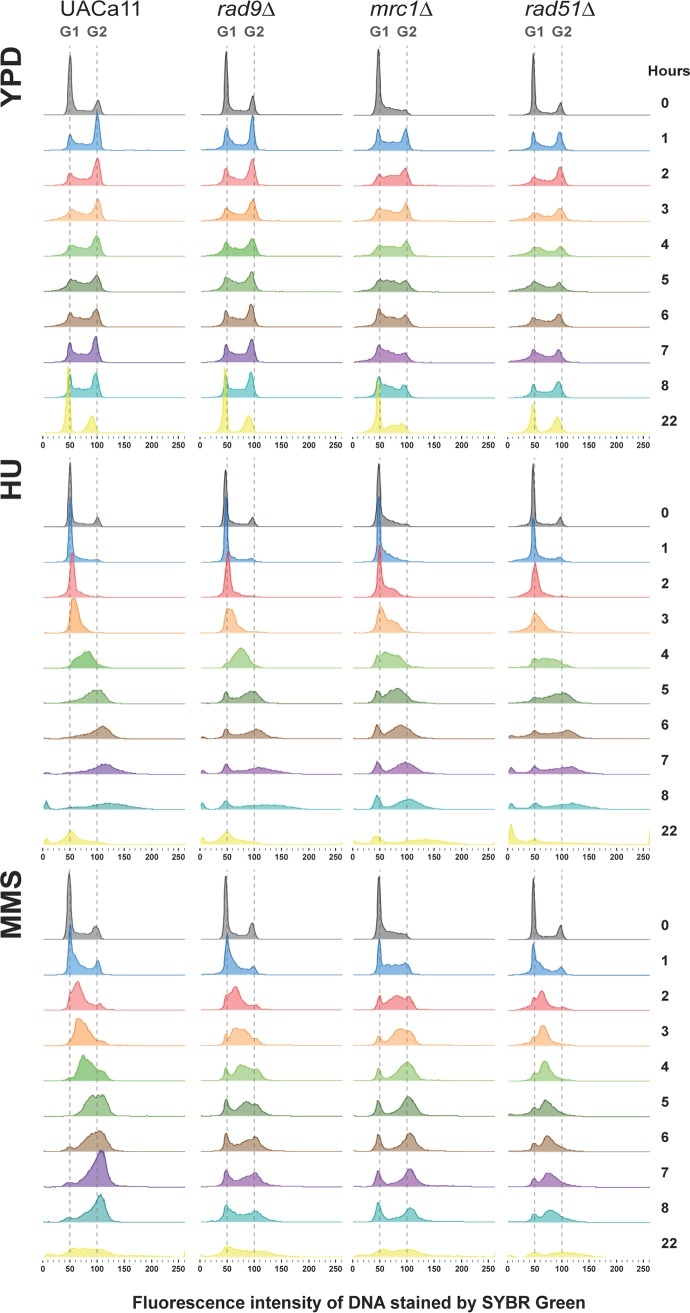
Cell cycle progression of Candida auris mutants under genotoxic stress. Histograms show cell cycle profiles obtained by flow cytometry of the wild type (UACa11) and the indicated mutant derivatives. Cells, previously arrested in G_1_ by nitrogen starvation, were transferred to fresh YPD broth and grown for 165 min at 30°C to restart the cycle before addition of 100 mM HU, 0.02% MMS, or no drug (time point 0 h). Cells were harvested at the indicated time points and DNA stained using SYBR green I. The amount of DNA is expressed as fluorescence intensity. Approximate positions of G_1_ and G_2_ peaks are indicated with dotted lines.

The S phase checkpoint slows down the cell cycle in response to DNA damage or DNA replication inhibition. The role of C. auris Rad51, Rad9, and Mrc1 for cell cycle arrest in response to genotoxic stress was further studied. Wild-type C. auris (UACa11) cultures can be arrested in G_1_ by nitrogen starvation; almost 100% of cells grown without a nitrogen source were arrested in G_1_ after 7 h and remained in G_1_ during prolonged (24 h) starvation (see Fig. S8A at https://doi.org/10.6084/m9.figshare.11378550). Upon return to favorable growth conditions (YPD), wild-type cultures had a lag phase of ∼2.5 to 3 h (Fig. S8B). Therefore, we grew wild-type and mutant yeast cultures for 165 min in YPD medium after G_1_ arrest before we started the experiments (time point 0 h) by adding 100 mM HU or 0.02% MMS as a genotoxic challenge; no drugs were added for the control. Cells were harvested at different time points, and the replication status of the cells was determined by flow cytometry (Fig. 4). Under unperturbed conditions, wild-type (UACa11), *rad9*Δ, and *rad51*Δ cells progressed from G_1_ to G_2_ within the first hour after reentering the cell cycle (Fig. 4). This is in line with these mutants showing wild-type growth in solid medium in the absence of genotoxins (Fig. 3). In *mrc1*Δ cells a larger fraction of the cell population was in S phase, which suggests that S phase might last longer in this mutant, which is similar to results in S. cerevisiae and C. albicans (20, 40, 41). Intriguingly, in C. auris this does not result in a notable growth defect on solid medium (Fig. 3).

In the parental wild-type strain, the S phase checkpoint is functional, and in the presence of HU and MMS cell cycle progression is slower than that under unperturbed conditions, with a large fraction of cells in S phase between 4 and 6 h (Fig. 4). Eventually, the checkpoint adapts, and cells move to G_2_ (Fig. 4). This is also largely true for *rad9*Δ and *rad51*Δ cells in the presence of HU, indicating a minor role in the HU response (Fig. 4). The *mrc1* null mutant behaves differently and shows a slower progression than the parental strain since after 6 h the majority of cells are still in S phase (Fig. 4). In the *rad51*Δ mutant most of the cells were arrested in S phase under MMS treatment, whereas cell cycle progression in the *rad9*Δ and *mrc1*Δ mutants was similar to that of the parental strain under these conditions (Fig. 4). However, in all the mutants, but not the wild type, a small population of G_1_ cells was always present during genotoxin treatments (Fig. 4). This could mean that some cells never enter the cell cycle after the G_1_ arrest or that they escape the S phase checkpoint early on and progress through the cell cycle to G_1_ quickly; these possibilities are not mutually exclusive. Indications that the latter possibility actually occurs are (i) that these G_1_ populations grow over time in *rad9*Δ and *mrc1*Δ mutants under both genotoxic conditions and (ii) that it also occurs in the wild type when cells are challenged with MMS. This indicates that *rad9*Δ and *mrc1*Δ cells are indeed defective in the S phase checkpoint. At 22 h the cycle seems to be partially restored (Fig. 4) though the presence of filaments and increased cell death after genotoxic treatment confound the interpretation of this result. Overall, these results support the idea that a functional S phase checkpoint exists in C. auris.

### The S phase checkpoint is involved in pseudohyphal growth.

**FIG 5:**
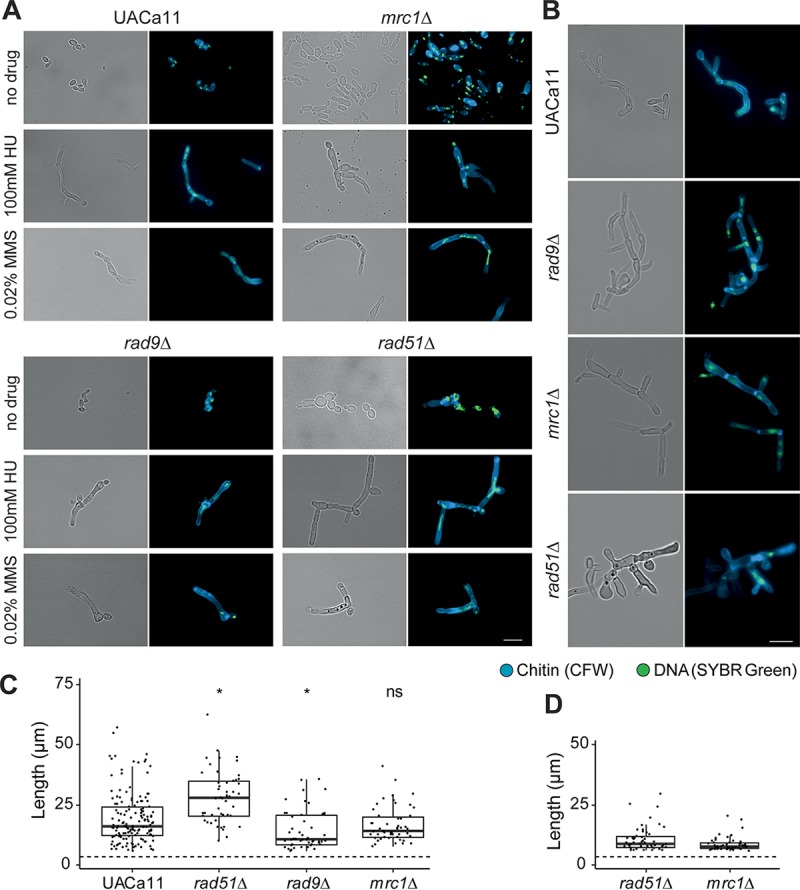
Microscopic analysis of filament formation in Candida auris *rad9*, *mrc1*, and *rad51* mutants (UACa11 background). (A) Representative microscopy images of C. auris filaments after growth of wild-type (UACa11), *rad9*Δ, *mrc1*Δ, and *rad51*Δ cells in YPD broth with or without the addition of the indicated drug for 18 to 20 h at 30°C. (B) Representative microscopic images of C. auris filaments after growth of wild-type (UACa11), *rad9*Δ, *mrc1*Δ, and *rad51*Δ cells on YPD plates containing 100 mM HU after 3 days at 30°C. In panels A and B, bright-field images are shown in the left columns, and merged fluorescent images (chitin stained by calcofluor white [CFW] and DNA stained by SYBR green I) are shown in the right columns. Scale bar, 10 μm. (C) Length of filaments observed after growth of wild-type (UACa11) (*n* = 150), *rad51*Δ (*n* = 50), *rad9*Δ (*n* = 50), and *mrc1*Δ (*n* = 50) cells in YPD broth containing 100 mM HU for 18 to 20 h at 30°C. Only cells longer than 6 μm were considered filaments. The dotted line indicates average length of 300 yeast cells (wild-type UACa11 grown in YPD for 18 to 20 h at 30°C). *, *P < *0.05, for results relative to those of the wild type (UACa11) (Wilcoxon rank sum test); ns, not significant. (D) Length of filaments observed after growth of *rad51*Δ (*n* = 50) and *mrc1*Δ (*n* = 50) cells in YPD medium for 18 to 20 h at 30°C without genotoxic stress. Only cells longer than 6 μm were considered filaments. The dotted line indicates average length of 300 yeast cells (wild-type UACa11 grown in YPD for 18 to 20 h at 30°C).

HU and MMS treatments induce pseudohyphal growth in C. albicans and S. cerevisiae which is dependent on activation of the S phase checkpoint because a reduction of filamentation was described for strains carrying mutations in the gene coding for the S phase checkpoint kinase Rad53 (19, 20). The ability of mutant C. auris to produce filaments was tested in liquid medium (Fig. 5A; see Fig. S1 at the URL mentioned above) and on solid medium (Fig. 5B; Fig. S2). As described above, wild-type cells of strain UACa11 form pseudohyphal filaments upon treatment with various genotoxins (Fig. 1).

C. auris *rad9*Δ cells grew as yeasts without genotoxic stress and as pseudohyphae in the presence of HU, similar to growth of the wild type (Fig. 5) and of C. albicans *rad9*Δ (20). Filaments formed in C. auris *rad9*Δ are significantly shorter than those of the wild type. Intriguingly, *rad9*Δ cells produced filaments growing in liquid medium containing MMS (Fig. 5; see Fig. S1 at the URL mentioned above), as observed in the wild-type parent, but failed to form filaments on solid medium with MMS even after 2 days (Fig. S2). That partially contrasts with the situation in C. albicans, where *rad9*Δ cells were somewhat compromised for filamentous growth in response to MMS compared to those of the wild type but would still form short pseudohyphae (20). After 3 days on solid medium containing 0.02% MMS, a noticeable fraction of *rad9*Δ cells became very large and round (giant cells); most of these giant cells are gone after 7 days of culture (presumably because giant cells have a low viability) (Fig. S2). These giant cells were also observed in the wild type and other mutants, especially under genotoxic stress, although at much lower frequency (Fig. S1 and S2), indicating that the lack of Rad9 enhances the production of this cell type.

C. auris *mrc1*Δ cells were elongated but not fully filamented after growth of 18 to 20 h in liquid medium without genotoxins, in contrast to the round or oval shape of wild-type cells (Fig. 5). This differs somewhat from growth of C. albicans where Mrc1-defective cells displayed pseudohyphal growth without stress (20). However, on solid medium without genotoxic stress, pseudohyphae were observed in *mrc1*Δ cells (see Fig. S2 at the URL mentioned above). In the presence of HU or MMS, the phenotype of the *mrc1*Δ mutant was almost identical to that of the wild-type parent in both liquid and solid media.

As observed in *rad52*Δ and *rad51*Δ cells in C. albicans (37, 42), a sizeable fraction of cells in the C. auris *rad51*Δ mutant showed constitutive pseudohyphal growth under unstressed conditions (Fig. 5; see Fig. S1 and S2 at the URL mentioned above). The remainder of the cells showed a yeast phenotype or were somewhat elongated and often of aberrant shape (e.g., giant cells or very wide and elongated cells). When treated with HU or MMS, this mutant formed pseudohyphae to a similar extent as the wild type, but the filaments tended to be longer (Fig. 5C).

Overall, this suggests a role of the S phase checkpoint in C. auris filamentous growth although the involvement of this pathway slightly differs from what has been observed in C. albicans.

### C. auris filamentation is strain dependent.

**FIG 6:**
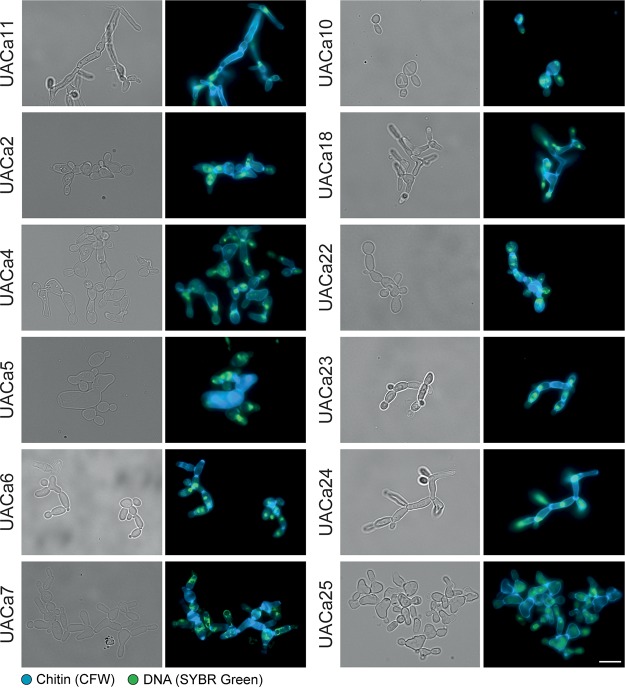
Different degrees of filamentation in Candida auris clinical isolates. Representative microscopy images of selected clinical isolates (see Table S2 at https://doi.org/10.6084/m9.figshare.11378550) after growth in YPD broth containing 100 mM HU for 18 to 20 h at 30°C. Bright-field images are shown in the left columns, and merged fluorescent images (chitin stained by calcofluor white [CFW] and DNA stained by SYBR green I) are shown in the right columns. Scale bar, 10 μm.

Strikingly, filamentation in C. auris was strain dependent. We tested the filamentation of 22 different C. auris clinical isolates (see Table S2 at https://doi.org/10.6084/m9.figshare.11378550) covering the four major clades (43) by treatment with HU (Fig. 6; Fig. S9). Among them, different grades of filamentation were observed: some strains showed longer filaments (UACa11 or UACa24) than others (UACa6 or UACa23); some were straight and thin (UACa11 or UACa24) or wider and shorter, growing as chains of (elongated) cells (bubbles) (UACa7 or UACa22); some formed aberrant cell shapes more frequently (UACa4 or UACa25). The clade III strain UACa10 seems to be unable to produce filaments when treated with genotoxins; it tended to produce bigger cells than in those produced under unperturbed conditions, however. The grade of filamentation was not obviously correlated with a particular clade since isolates from the same clade showed different phenotypes.

To evaluate if these strain-dependent filamentation phenotypes are due to differences in cell cycle progression under genotoxic stress, we selected five strains (UACa10, UACa11, UACa18, UACa22, and UACa25) representing all four clades and showing different grades of filamentation (Fig. 6). As before, these strains were subjected to nitrogen starvation to arrest them in G_1_, and after pregrowth in liquid medium for 165 min, genotoxins were added. In the presence of HU, the cell cycle in strains UACa10, UACa22, and UACa25 progressed ∼1 h faster than the cell cycle in strain UACa11 (see Fig. S10 at the URL mentioned above). When UACa25 cells were treated with MMS, the cell cycle progression in was ∼2 h faster (Fig. S10). In the slow-growing clade II isolate UACa18, a fraction of the cell population remained in G_1_ after MMS treatment (Fig. S10), which indicates that the restart of the cell cycle after G_1_ arrest might be slower in this strain. Although we observed some subtle differences between the C. auris isolates, these do not explain their substantial differences in filamentation.

Therefore, we looked for single nucleotide polymorphisms (SNPs) between two strains from the same clade showing different grades of filamentation, which might explain this differential cellular behavior (isolates from different clades are too diverse, and it would be too difficult to filter out the noise from these data) (43–45). We selected two strains from clade I (South Asia), UACa1 and UACa4, with different capabilities of forming pseudohyphae, which differ by 298 SNPs. Intriguingly, of a total of 99 nonsynonymous SNPs within open reading frames, we found 23 genes with a predicted/demonstrated role in filamentation and one hypothetical protein of unknown function with an SNP causing a premature stop codon in UACa1 (see Table S3 at the URL mentioned above). Unfortunately, this makes it unfeasible to determine a single target gene as the reason for the phenotypic differences between these two strains and suggests that several pathways could be involved in enabling or preventing a particular strain from forming filaments under stress conditions.

## DISCUSSION

Due to the recent emergence of C. auris, there is a lack of understanding about its life cycle, which impedes full comprehension of its origin, cellular behavior, and pathogenic traits. Due to the evolutionary distance to the best-studied *Candida* species, C. albicans (30), inferences from research on C. albicans are not transferable to C. auris. This certainly is the case for the morphological switch between yeast cells and filaments. Most cues causing filamentation in C. albicans do not work in C. auris (15; this study). Accordingly, several genes essential for filamentation in C. albicans and S. cerevisiae, such as *EED1*, *FLO11*, *HWP1*, *HWP2*, or *ECE1* (11, 12) are missing from the C. auris genome (see Table S1; all supplemental figures and tables can be found at https://doi.org/10.6084/m9.figshare.11378550) (30), and some important regulatory determinants of filamentation, such as Ume6, show conspicuous differences between C. auris and C. albicans (Fig. S3). Accordingly, *tup1*Δ cells do not trigger constitutive filamentation in C. auris (Fig. 2A) as they do in C. albicans (34). However, untreated *tup1*Δ cells showed defective cell separation. This phenotype might be due to overexpression of *HGC1*, encoding a G_1_ cyclin, which controls the expression of septum-degrading enzymes in C. albicans (46), and its expression is kept off by the repressor complex Tup1/Nrg1 (47). Altogether, these results indicate that C. auris is incapable of inducing filamentous growth under the same stimuli as C. albicans.

However, we observed that genotoxic stress triggers filamentation in most C. auris isolates tested (Fig. 1 and 6; see Fig. S9 at the URL mentioned above). These filaments displayed characteristics attributed to pseudohyphae (reviewed in references 9 and 11), as previously described in the presence of HU (48). Indeed, the presence of genotoxic drugs, such as MMS or HU, also triggers pseudohyphal growth in C. albicans and S. cerevisiae (18–20, 49, 50).

In fungi, the switch from yeast to filamentous growth is signaled through the mitogen-activated protein kinase (MAPK) and the fungal cyclic AMP (cAMP)-protein kinase A (PKA) pathways. Additionally, other pathways, such as the sucrose-nonfermentable (SNF), TOR, Hog1, and Rim101 pH-sensing pathways, influence filamentation (reviewed in references 11 to 13). This process involves ∼700 genes for pseudohypha formation in S. cerevisiae (27, 28) and more than 2,000 genes in C. albicans filamentous growth (29). However, mutation of key genes regulating filamentous growth, such as *HGC1*, *UME6*, *FLO8*, *TEC1*, *NRG1*, *CPH1* (*STE12* in S. cerevisiae), or *EFG1*, does not affect HU-induced pseudohypha formation (19, 49, 50). Therefore, pseudohyphal growth upon genotoxic stress apparently involves, at least partially, different mechanisms.

Genotoxic drugs can induce a variety of DNA damage types and/or perturbation of DNA replication forks, thus triggering the S phase checkpoint. As part of the checkpoint response, the kinase Rad53 is activated, which leads to a temporal cell cycle delay until the issue is resolved and the cell cycle can continue (reviewed in reference 17). A delay of the cell cycle in S phase and a subsequent transient arrest in G_2_/M were observed in various C. auris strains in the presence of the genotoxic agents HU and MMS (Fig. 4; see Fig. S10 at the URL mentioned above); this suggests that C. auris has a functional S phase checkpoint. Furthermore, mutation of the S phase checkpoint genes *MRC1* and *RAD9* leads to a defective cell cycle arrest since some cells seem to progress quickly to G_1_ under genotoxic stress (Fig. 4), similar to growth of the same C. albicans or S. cerevisiae mutants (20, 38, 40, 41, 51). Although activation of Rad53 by DNA damage or perturbed DNA replication forks is sensed differently, both types of DNA lesions possess the molecular signal that triggers the response: an accumulation of single-stranded DNA (ssDNA) which acts as a signal for recruiting and later activating Mec1 (52–54). Homologous recombination is required to repair the DNA lesions generated under genotoxic stress, and, therefore, Rad51 and Rad52 are required to prevent an excess of ssDNA (52, 55, 56). Indeed, in C. auris *rad51*Δ, cells were arrested in S phase during MMS treatment, which suggests an inability to restore the DNA lesions and, therefore, a constitutive activation of Rad53. Moreover, similar to the *rad9*Δ or *mrc1*Δ strain and in contrast to the wild type, a fraction of the genotoxin-stressed *rad51*Δ cell population seems unable to restart the cell cycle after the G_1_ arrest or move through the cell cycle without delay (see above) (Fig. 4). Altogether, our results suggest that, similar to other ascomycetes, C. auris has fully functional S phase checkpoint and homologous recombination pathways.

Pseudohyphal growth in response to genotoxins is S phase checkpoint dependent as *rad53* mutants in C. albicans or S. cerevisiae and *mec1*Δ mutants in S. cerevisiae showed a drastic decrease of filamentation under genotoxin treatment (19, 20). Similarly, strains defective for *RAD9* or *MRC1* also showed alterations of morphology in the presence of genotoxic stress in C. albicans (20). After MMS treatment, a C. albicans *RAD9*-defective mutant forms filaments which are considerably shorter than those in the wild type. In contrast, filamentation of a C. auris *rad9*Δ mutant was not different from that of the wild type after 24 h of MMS treatment in liquid culture (see Fig. S1B at the URL mentioned above). However, on plates, fewer pseudohyphae were observed in the C. auris *rad9*Δ mutant than in the wild type after long-term exposure to MMS (>2 days), albeit a higher proportion of yeast cells became enlarged in the mutant (Fig. S1A and S2). This cell enlargement was obvious in, but not restricted to, *rad9*Δ cells and could occasionally be observed in the wild type and other mutants, especially in the presence of MMS (Fig. S1A and S2). In other fungi, the presence of enlarged round cells has been described as Titan cells in Cryptococcus neoformans (57, 58) or Goliath cells in C. albicans (59). Further studies will be necessary to elucidate whether these large cells in C. auris resemble Titan or Goliath cells or are something completely different. Taking these observations together, this would suggest that in C. albicans Rad9 might control different or additional HSGs than those controlled by C. auris (see below). The C. auris *mrc1*Δ mutant induced, at least partially, pseudohyphal growth in the absence of any genotoxic stress; otherwise, its growth was indiscernible from that of the wild type (Fig. 5; Fig. S1 and S2). This result is similar to observations in C. albicans (20, 60). Interestingly, a C. albicans *SGS1* mutant strain, which fails to activate Rad53 via Mrc1, forms filaments under unperturbed conditions (61). As a possible explanation, formation of DNA double-strand breaks in *mrc1*Δ strains and the subsequent activation of Rad53 through Rad9 have been suggested (40, 62). An S phase-independent role, however, has also been described for Mrc1, regulating the replication initiation through interaction with Cdc7, a conserved kinase that triggers firing at each replication origin (63), which regulates the mitotic exit through interaction with Cdc5 (64). This could explain the filamentation observed in the *mrc1*Δ mutant (see below) and the larger S phase population observed in this mutant under unperturbed conditions (Fig. 4). Mutation of homologous recombination genes such as *RAD51*, *RAD52*, *MRE11*, or *RAD50* causes constitutive pseudohypha formation in C. albicans (37, 42, 61). We observed that deletion of *RAD51* and *RAD57* also triggered constitutive pseudohyphal growth in C. auris (Fig. 5; see also Fig. S1, S2, and S7 at the URL mentioned above).

The S phase checkpoint is a complex process, including several back-up mechanisms which could explain why *MRC1* and *RAD9* mutants are still able to arrest the cell cycle and produce filaments under genotoxic stress. Indeed, it has been reported that Mrc1 depends on Rad9 to stay activated for a long period and that Rad53 is rapidly but only transiently activated by Mrc1 in *rad9*Δ cells and is slowly, but continuously, activated by Rad9 in the absence of *MRC1* (17, 51). Accordingly, the double mutant *mrc1*Δ *rad9*Δ strain is inviable (40, 62). That would explain our observations, under MMS treatment, that in the short term C. auris *rad9*Δ produces filaments, due to activation of Rad53 by Mrc1; but in the long term, Mrc1 is not able to maintain this activation, and cells lose their filamentation phenotype.

**FIG 7:**
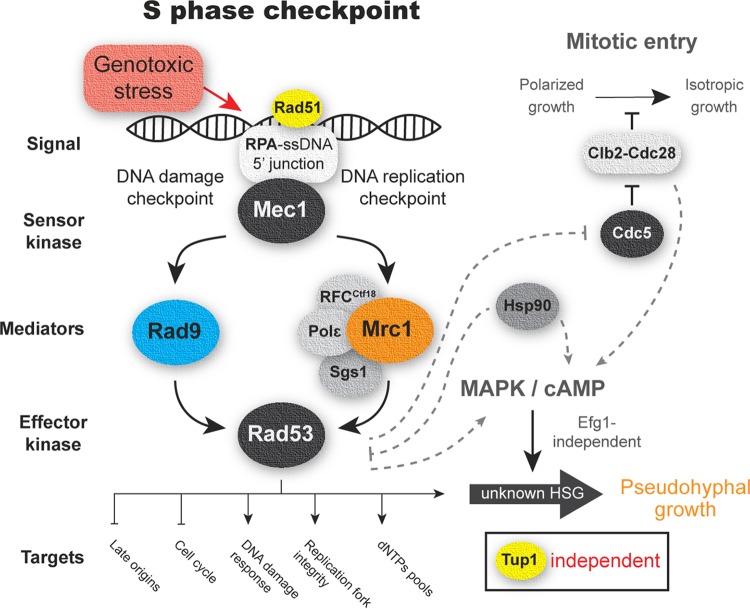
Schematic representation S phase checkpoint expected for Candida auris. A working model of the S phase checkpoint based on results of this study and related literature in yeast is shown. Proteins tested in this study are in color. Possible S phase checkpoint downstream pathways related to filamentous growth are connected by dotted lines. Tup1 is a major negative regulator of filamentation in C. albicans; filamentation in C. auris is unaffected by deletion of *TUP1*. HSGs, hyphal-specific genes.

Mechanisms involved in pseudohyphal growth in response to S phase checkpoint activation are not well understood, and further studies will be necessary. However, one mechanism could involve the constitutive activation of the Clb2-Cdc28 complex by Rad53 in response to genotoxic stress through the polo kinase Cdc5 (65–67). The activation of Clb2-Cdc28 prevents the entry into mitosis and the associated switch from polarized to isotropic growth (68, 69); therefore, cells would be stuck in the apical growth phase, thus forming filaments. Hence, mutation of *CLB2*, *CDC28*, and *CDC5* triggers constitutive pseudohyphal growth in yeasts (49, 70, 71). Furthermore, the cAMP and MAPK pathways have been implicated in pseudohyphal growth in response to genotoxic stress via downstream regulators (18, 42, 50, 72). A plausible model for C. auris is depicted in Fig. 7; our model also takes into account that, in C. auris, constitutive pseudohyphal growth is triggered by downregulation of *HSP90*, a heat shock family protein, which acts as a chaperone and influences a diverse range of signal transducers (48). Inhibition of *HSP90* induces pseudohyphal growth via cAMP-PKA signaling in an Efg1-independent way in C. albicans (73), and, interestingly, a direct inhibition of Rad53 by Hsp90 has been observed in S. cerevisiae (74).

During infection, cells may encounter various conditions that lead to cell cycle arrest, produced either by the host or by other microorganisms cohabiting a given niche. Switching to filamentous growth might be advantageous, allowing cells to escape from such perturbations. However, to our knowledge, filamentous growth in the context of pathogenesis and colonization has only been described once in C. auris (21). This strain recovered from an infected mouse displayed filamentous growth at temperatures below 25°C. However, none of our strains have developed filamentation under these conditions. In any case, there currently are not enough data available for C. auris infection to fully appreciate the potential role of morphogenetic switching, which contributes to full virulence in C. albicans (reviewed in references 11, 13, and 14). The different capacities of different strains to form filaments (21; this study) and the plasticity of the C. auris genome (43, 75) are indications of the flexibility and adaptability of this fungus, suggesting that different clinical isolates could use morphogenetic switching during different phases of pathogenesis.

## MATERIALS AND METHODS

### Yeast strains and culture conditions.

*Candida* strains used in this study are listed in Table S2 in the supplemental material (all supplemental figures and tables can be found at https://doi.org/10.6084/m9.figshare.11378550). Yeast cells were grown at 30°C on YPD plates (1% yeast extract, 2% mycological peptone, 2% glucose, 2% agar; Oxoid, Basingstoke, UK) or with shaking at 200 rpm in YPD broth (same constituents as plates, but without agar). Cell concentrations were determined by measuring the optical density of the culture at a wavelength of 600 nm (OD_600_) on an Ultraspec 2000 (Pharmacia Biotech, Uppsala, Sweden) spectrometer (calibration defined an OD_600_ of 1 to contain ∼3 × 10^7^ C. auris cells/ml).

Spot assays were carried out on YPD agar adding the indicated drug where necessary (Fig. 3; see also Fig. S7A). Cell concentrations were determined by OD_600_ after overnight growth in YPD broth, and four 1:10 serial dilutions containing 10^2^ to 10^5^ cells were plated and grown at 30°C for 3 days (unless indicated otherwise).

For filament visualization, ∼1 × 10^7^ cells were grown for 18 to 20 h in YPD broth, unless indicated otherwise, with addition of 100 μM HU, 0.02% MMS, or 5 μg/ml 5-FC. Other potential filamentation conditions tested were the following: Lee’s medium at pH 3.5 and pH 6.5 (76); YPD (see above) or RPMI 1640 medium containing 20% fetal calf serum (Sigma-Aldrich Corp., St. Louis, MO); YPD medium at a temperature of 25, 30, or 37°C; presence of 0.5% isoamyl alcohol in YPD medium; and 25 μg/ml Bleocin in YPD medium. To check filamentation on solid medium, fresh cells were streaked on YPD plates containing 100 μM HU, 0.02% MMS, or 5 μg/ml 5-FC and grown at 30°C for the times indicated (Fig. 1 and 5; see also Fig. S2).

### Cell cycle analysis and microscopy.

To synchronize cultures in G_1_, cells were grown in YPD broth overnight at 30°C and 200 rpm. Then 1 × 10^7^ cells were washed with sterile water and transferred to nitrogen starvation conditions (6.7 g/liter yeast nitrogen base [YNB] without amino acids and without ammonium sulfate [Sigma-Aldrich] and 20 g/liter glucose) and grown at 30°C and 200 rpm. Samples were harvested by centrifugation (1,000 × *g*, 2 min) and fixed by addition of 70% ethanol (EtOH) at the indicated time points (Fig. 4; see also Fig. S8 and S10). Cells were stored at +4°C before proceeding to flow cytometry (see below).

The cell cycle of cells previously arrested in G_1_ from different C. auris strains in the presence of genotoxic drugs was determined. For this purpose, 1 × 10^7^ cells, obtained after growth in YPD broth overnight at 30°C and 200 rpm, were harvested, washed with sterile water, and transferred to nitrogen starvation conditions (see above) for 15 to 17 h at 30°C and 200 rpm. Then, 1 × 10^8^ cells were transferred to 10 ml of fresh YPD broth. To restart the cell cycle, cells were grown for 165 min at 30°C and 200 rpm, after which 100 mM HU or 0.02% MMS were added (a third culture without any additions served as a control). Cells were harvested by centrifugation (1,000 × *g*, 2 min)and fixed by addition of 70% ethanol every hour from 0 to 8 h and at a final time point of 22 h. Cells were stored at +4°C before proceeding to flow cytometry (see below). For flow cytometry, yeast samples were prepared as previously described (75, 77).

For microscopy, cells were fixed by addition of 1 ml 70% EtOH and incubated for at least 2 h at 4°C. Fixed cells were pelleted (2 min at 1,000 × *g*) and resuspended in sodium citrate buffer (14.7 g/liter sodium citrate, pH 7.5). Cells were then treated with RNase A (250 μg per 1 × 10^7^ cells) and proteinase K (1,000 μg per 1 × 10^7^ cells) for 1 h at 50°C; subsequently, Triton X-100 (Sigma-Aldrich) was added to a final concentration of 0.25%. Cells were stained for DNA (SYBR green I, 1:5000; Sigma-Aldrich) and for chitin (1 μg/ml calcofluor white; Sigma) at +4°C overnight. For time course experiments, the same samples were used for flow cytometry and microscopy (adding 1 μg/ml calcofluor white for microscopy). Rhodamine-phalloidin staining was carried out as previously described (78) with a few modifications. Approximately 1 × 10^7^ cells from an overnight culture were fixed for 1 h in 4% formaldehyde (phosphate-buffered saline [PBS] buffered) at room temperature, harvested by centrifugation (2 min at 1,000 ×*g*), and resuspended in PBS before addition of 10 μl of rhodamine-phalloidin (6.6 μM in methanol) and 1 μl of 4′,6′-diamidino-2-phenylindole (DAPI; 10 μg/ml). Samples were incubated in the dark for 1 h and washed in PBS (2 min, 1,000 × *g*) twice. Finally, cells were resuspended in ProLong Diamond antifade (Thermo Fisher Scientific, Waltham, MA).

Fluorescent images were taken on a Zeiss Axio Imager M2 microscope with a Zeiss 503 camera and analyzed using ZEN 2 blue edition software (Carl Zeiss Microscopy, Jena, Germany). Standard bright-field microscopy was performed on a BX50 microscope (Olympus, Tokyo, Japan) equipped with an Infinity 1 camera (Lumenera, Ottawa, Canada) and analyzed using the Infinity capture software, version 6.5.7 (Lumenera, Ottawa, Canada). Colony morphology was imaged on a Zeiss Stemi 2000-c binocular (Carl Zeiss Microscopy, Jena, Germany) equipped with an Infinity 1 camera (Lumenera, Ottawa, Canada).

Phenotype quantifications and filament length measurements were done from bright-field microscopy images using the cell counter and the measuring tools included in ImageJ (version 1.47; National Institutes of Health, Bethesda, MD). Box plots, bar charts, and statistical analysis were done in R (version 3.6.0) (https://www.r-project.org/) using the ggplot2 and the ggpubr libraries in the RStudio environment (version 1.2.1335; RStudio, Inc., Boston, MA). Kruskal-Wallis and Wilcoxon rank sum tests at an alpha level of 0.05 were used to identify statistical differences between data sets.

### Nucleic acid manipulations.

For fast genomic DNA (gDNA) extraction, cells from a single colony were suspended in 20 mM NaOH, boiled at 100°C for 10 min, and incubated on ice for 10 min. Samples were then centrifuged (2 min at 1,000 × *g*), and a small amount of supernatant was used for PCRs.

For high-quality gDNA extractions, cells from overnight cultures were harvested (2 min at 1,000 × *g*) and washed with double-distilled water (ddH_2_O). After the supernatant was removed, acid-washed glass beads (Sigma-Aldrich) and DNA extraction buffer (1 M NaCl, 2% Triton x-100, 1 M Tris-HCl, pH 8, 0.5 M EDTA, 1% SDS) were added. Cells were disrupted in a Vibrax VXR (IKA GmbH & Co. KG, Staufen, Germany) multitube mixer for 3 min before addition of 1 volume of phenol-chloroform-isoamyl alcohol (25:24:1) (Sigma-Aldrich). Samples were mixed vigorously and centrifuged (10 min at 12,000 × *g*). The supernatant was transferred to a fresh reaction tube, and 0.1 volume of 3 M sodium acetate, pH 5.2, and 2 volumes of ice-cold 100% ethanol were added. After precipitation (30 to 60 min at –20°C), samples were centrifuged (5 min at 12,000 × *g*) and washed with 70% ethanol. Pellets were airdried and resuspended in ddH_2_O, and 5 μl of RNase (10 mg/ml) was added, followed by an incubation of 30 to 60 min at 37°C. A small amount of a 1:10 dilution was used for subsequent PCRs.

Standard PCRs were carried out using DreamTaq PCR Master Mix (Thermo Fisher Scientific) according to the manufacturer’s instructions. All primers used in this study (see Table S4 at https://doi.org/10.6084/m9.figshare.11378550) were designed using the C. auris reference genome (https://www.ncbi.nlm.nih.gov/genome/?term=txid498019) and synthesized by Sigma-Aldrich.

### Gene deletion constructs and transformation.

All gene deletion constructs were amplified using Phusion High-Fidelity PCR Master Mix (Thermo Fisher Scientific) as recommended by the supplier.

Genes were deleted using the nourseothricin resistance marker *CaNAT1* flanked by 1.5- to 2-kb regions of homology to up- and downstream sequences of the target gene. *CaNAT1*, including *TEF* promoter and terminator sequences, was PCR amplified from pV1025 (79) using oligonucleotides oUA315 and oUA316 (see Table S4 at the URL mentioned above) and cloned into a BamHI-linearized pFA6a backbone by NEBuilder DNA Assembly Master Mix (New England BioLabs, Ipswich, MA). The assembly mix was transformed into NEB 10-beta Escherichia coli cells (New England BioLabs) according to the manufacturer’s instructions. The resulting plasmid, pALo218, was then used to amplify *CaNAT1* with the oligonucleotide oUA353 and oUA354 (Table S4). Up- and downstream homologous regions of the target gene were PCR amplified from gDNA of UACa11 (Table S2) using specific primers for each target gene (Table S4). The oligonucleotide primers on the 3′ end of the upstream region and at the 5′ end of the downstream region include short homologies to the *CaNAT1* marker to assemble transformation cassettes via fusion PCR (80). The three PCR products (*CaNAT1* and up- and downstream homology regions) were combined in roughly equimolar amounts (∼0.15 pmols) together with Phusion High-Fidelity PCR Master Mix (Thermo Fisher Scientific) containing 2.5% dimethyl sulfoxide (DMSO) and subjected to eight PCR cycles (10 s at 98°C, 30 s at 55°C, and 60 s at 72°C). Nested primers (see Table S4 at the URL mentioned above) under standard PCR conditions were used to amplify the final transformation cassettes.

Gene deletion constructs were transformed into C. auris strain UACa11 using a protocol developed for C. albicans (81), with small modifications. Briefly, cells from a culture grown in YPD medium to stationary phase were diluted 1:100 into fresh YPD broth and incubated with shaking at 30°C until they reached mid-exponential phase (OD_600_ of 0.5 to 0.8). Cells were centrifuged (2 min, 1,000 × *g*), washed once with ddH_2_O, and resuspended in 100 mM lithium acetate. Transformation mixtures contained ∼1 μg of DNA of the transformation cassette, 100 μg of carrier DNA (herring sperm DNA solution; Thermo Fisher Scientific), 37% polyethylene glycol (PEG) 3350, 100 mM lithium acetate, and ∼1.5 × 10^8^ C. auris cells. The transformation mixture was incubated overnight (16 to 20 h) at 30°C. After a heat shock of 15 min at 44°C, cells were pelleted by centrifugation (2 min at 1,500 × *g*), resuspended in YPD medium, and incubated with shaking for 4 h at 30°C. Cells were then plated onto selective YPD agar containing 100 μg/ml nourseothricin (clonNAT; Werner BioAgents GmbH, Jena, Germany) and incubated at 30°C until transformants appeared. Gene deletion and correct integration were confirmed by PCR using primers located within the ORF of the target gene and primers located within and without the transformation cassette (see Fig. S5 and Table S4 at the URL mentioned above).

### *In silico* analysis.

Gene and protein sequences were obtained from the C. auris reference genome (assembly Cand_auris_B11221_V1) in the NCBI database (https://www.ncbi.nlm.nih.gov/genome/?term=txid498019). Interestingly, *RAD57* was not present in this reference genome but could be found in the draft genome of strain Ci6684 (82). Protein sequences of *Candida* spp. were obtained from the *Candida* Genome Database (http://www.candidagenome.org/) or from Uniprot (https://www.uniprot.org/), and the *Saccharomyces* Genome Database (https://www.yeastgenome.org/) served as source for S. cerevisiae protein sequences. Homology scores of C. auris proteins against C. albicans and S. cerevisiae proteins (E value, length aligned; identities are shown in Table S1, available at the URL mentioned above) were generated using the BLAST tools of the *Candida* or the *Saccharomyces* Genome Databases. Alignments were carried out by the MSAprobs method (default settings) using Jalview, version 2.10.5 (83).

For SNP calling, Illumina sequencing reads from two clade I strains (UACa1 and UACa4) were aligned (Z. K. Ross, N. A. R. Gow, and A. Lorenz, unpublished results) using BWA-MEM, version 0.7.12 (84), and processed with Samtools, version 0.1.19, view, sort, rmdup, and index (85). SNPs were then detected using Pilon, version 1.22 (86), filtering the resulting variant call format (VCF) file for genotype 1/1 only. Low coverage (less than 10% of mean coverage), ambiguous positions, and deletions were removed. The reference genome was annotated with Augustus, version 3.3.1 (87), *ab initio* gene prediction software, and VCF annotator (http://vcfannotator.sourceforge.net) was used to predict the effect of the SNPs called on the annotated genes.

## References

[1] Satoh K, Makimura K, Hasumi Y, Nishiyama Y, Uchida K, Yamaguchi H 2009 *Candida auris* sp. nov., a novel ascomycetous yeast isolated from the external ear canal of an inpatient in a Japanese hospital. Microbiol Immunol 53:41–44. doi:10.1111/j.1348-0421.2008.00083.x. 19161556

[2] Kwon YJ, Shin JH, Byun SA, Choi MJ, Won EJ, Lee D, Lee SY, Chun S, Lee JH, Choi HJ, Kee SJ, Kim SH, Shin MG 2019 *Candida auris* clinical isolates from South Korea: identification, antifungal susceptibility, and genotyping. J Clin Microbiol 57:e01624-18. doi:10.1128/JCM.01624-18. 30728190 PMC6440790

[3] Rhodes J, Fisher MC 2019 Global epidemiology of emerging *Candida auris*. Curr Opin Microbiol 52:84–89. doi:10.1016/j.mib.2019.05.008. 31279224

[4] Lone SA, Ahmad A 2019 *Candida auris*—the growing menace to global health. Mycoses 62:620–637. doi:10.1111/myc.12904. 30773703

[5] Kean R, Ramage G 2019 Combined antifungal resistance and biofilm tolerance: the global threat of *Candida auris*. mSphere 4:e00458-19. doi:10.1128/mSphere.00458-19. 31366705 PMC6669339

[6] Forsberg K, Woodworth K, Walters M, Berkow EL, Jackson B, Chiller T, Vallabhaneni S 2019 *Candida auris*: the recent emergence of a multidrug-resistant fungal pathogen. Med Mycol 57:1–12. doi:10.1093/mmy/myy054. 30085270

[7] Jackson BR, Chow N, Forsberg K, Litvintseva AP, Lockhart SR, Welsh R, Vallabhaneni S, Chiller T 2019 On the origins of a species: what might explain the rise of *Candida auris*? J Fungi (Baseo) 5:E58. doi:10.3390/jof5030058. PMC6787658 31284576

[8] Casadevall A, Kontoyiannis DP, Robert V 2019 On the emergence of *Candida auris*: climate change, azoles, swamps, and birds. mBio 10:e01397-19. doi:10.1128/mBio.01397-19. 31337723 PMC6650554

[B9] Sudbery P, Gow N, Berman J 2004 The distinct morphogenic states of *Candida albicans*. Trends Microbiol 12:317–324. doi:10.1016/j.tim.2004.05.008. 15223059

[B10] Berman J 2006 Morphogenesis and cell cycle progression in *Candida albicans*. Curr Opin Microbiol 9:595–601. doi:10.1016/j.mib.2006.10.007. 17055773 PMC3552184

[11] Sudbery PE 2011 Growth of *Candida albicans* hyphae. Nat Rev Microbiol 9:737–748. doi:10.1038/nrmicro2636. 21844880

[B12] Cullen PJ, Sprague GF 2012 The regulation of filamentous growth in yeast. Genetics 190:23–49. doi:10.1534/genetics.111.127456. 22219507 PMC3249369

[13] Noble SM, Gianetti BA, Witchley JN 2017 *Candida albicans* cell-type switching and functional plasticity in the mammalian host. Nat Rev Microbiol 15:96–108. doi:10.1038/nrmicro.2016.157. 27867199 PMC5957277

[14] Thompson DS, Carlisle PL, Kadosh D 2011 Coevolution of morphology and virulence in *Candida Species*. Eukaryot Cell 10:1173–1182. doi:10.1128/EC.05085-11. 21764907 PMC3187052

[15] Wang X, Bing J, Zheng Q, Zhang F, Liu J, Yue H, Tao L, Du H, Wang Y, Wang H, Huang G 2018 The first isolate of *Candida auris* in China: clinical and biological aspects. Emerg Microbes Infect 7:93. doi:10.1038/s41426-018-0095-0. 29777096 PMC5959928

[16] Kron SJ, Gow NA 1995 Budding yeast morphogenesis: signalling, cytoskeleton and cell cycle. Curr Opin Cell Biol 7:845–855. doi:10.1016/0955-0674(95)80069-7. 8608015

[17] Pardo B, Crabbé L, Pasero P 2017 Signaling pathways of replication stress in yeast. FEMS Yeast Res 17:fow101. doi:10.1093/femsyr/fow101. 27915243

[18] Bachewich C, Nantel A, Whiteway M 2005 Cell cycle arrest during S or M phase generates polarized growth via distinct signals in *Candida albicans*. Mol Microbiol 57:942–959. doi:10.1111/j.1365-2958.2005.04727.x. 16091036

[19] Jiang YW, Kang CM 2003 Induction of *S. cerevisiae* filamentous differentiation by slowed DNA synthesis involves Mec1, Rad53 and Swe1 checkpoint proteins. Mol Biol Cell 14:5116–5124. doi:10.1091/mbc.e03-06-0375. 14565980 PMC284813

[20] Shi Q-M, Wang Y-M, De Zheng X, Lee RTH, Wang Y 2007 Critical role of DNA checkpoints in mediating genotoxic-stress–induced filamentous growth in *Candida albicans*. Mol Biol Cell 18:815–826. doi:10.1091/mbc.e06-05-0442. 17182857 PMC1805102

[21] Yue H, Bing J, Zheng Q, Zhang Y, Hu T, Du H, Wang H, Huang G 2018 Filamentation in *Candida auris*, an emerging fungal pathogen of humans: passage through the mammalian body induces a heritable phenotypic switch. Emerg Microbes Infect 7:188. doi:10.1038/s41426-018-0187-x. 30482894 PMC6258701

[22] Nyholm S, Thelander L, Gräslund A 1993 Reduction and loss of the iron center in the reaction of the small subunit of Mouse ribonucleotide reductase with hydroxyurea. Biochemistry 32:11569–11574. doi:10.1021/bi00094a013. 8218224

[23] Poli J, Tsaponina O, Crabbé L, Keszthelyi A, Pantesco V, Chabes A, Lengronne A, Pasero P 2012 dNTP pools determine fork progression and origin usage under replication stress. EMBO J 31:883–894. doi:10.1038/emboj.2011.470. 22234185 PMC3280562

[24] Vázquez MV, Rojas V, Tercero JA 2008 Multiple pathways cooperate to facilitate DNA replication fork progression through alkylated DNA. DNA Repair (Amst) 7:1693–1704. doi:10.1016/j.dnarep.2008.06.014. 18640290

[25] Grem JL 2000 5-Fluorouracil: forty-plus and still ticking. A review of its preclinical and clinical development. Invest New Drugs 18:299–313. doi:10.1023/a:1006416410198. 11081567

[26] Sherry L, Ramage G, Kean R, Borman A, Johnson EM, Richardson MD, Rautemaa-Richardson R 2017 Biofilm-forming capability of highly virulent, multidrug-resistant *Candida auris*. Emerg Infect Dis 23:328–331. doi:10.3201/eid2302.161320. 28098553 PMC5324806

[27] Jin R, Dobry CJ, McCown PJ, Kumar A 2008 Large-scale analysis of yeast filamentous growth by systematic gene disruption and overexpression. Mol Biol Cell 19:284–196. doi:10.1091/mbc.e07-05-0519. 17989363 PMC2174193

[28] Ryan O, Shapiro RS, Kurat CF, Mayhew D, Baryshnikova A, Chin B, Lin ZY, Cox MJ, Vizeacoumar F, Cheung D, Bahr S, Tsui K, Tebbji F, Sellam A, Istel F, Schwarzmüller T, Reynolds TB, Kuchler K, Gifford DK, Whiteway M, Giaever G, Nislow C, Costanzo M, Gingras AC, Mitra RD, Andrews B, Fink GR, Cowen LE, Boone C 2012 Global gene deletion analysis exploring yeast filamentous growth. Science 337:1352–1356. doi:10.1126/science.1224339. 22984072

[29] Azadmanesh J, Gowen AM, Creger PE, Schafer ND, Blankenship JR 2017 Filamentation involves two overlapping, but distinct, programs of filamentation in the pathogenic fungus *Candida albicans*. G3 (Bethesda) 7:3797–3808. doi:10.1534/g3.117.300224. 28951491 PMC5677161

[30] Munoz JF, Gade L, Chow NA, Loparev VN, Juieng P, Farrer RA, Litvintseva AP, Cuomo CA 2018 Genomic basis of multidrug-resistance, mating, and virulence in *Candida auris* and related emerging species. Nat Commun 9:5346. doi:10.1038/s41467-018-07779-6. 30559369 PMC6297351

[31] Lo W, Dranginis AM 1998 The cell surface flocculin Flo11 is required for pseudohyphae formation and invasion by *Saccharomyces cerevisiae*. Mol Biol Cell 9:161–171. doi:10.1091/mbc.9.1.161. 9436998 PMC25236

[32] Vinod PK, Sengupta N, Bhat PJ, Venkatesh KV 2008 Integration of global signaling pathways, cAMP-PKA, MAPK and TOR in the regulation of *FLO11*. PLoS One 3:e1663. doi:10.1371/journal.pone.0001663. 18301741 PMC2246015

[33] Younes SS, Khalaf RA 2013 The *Candida albicans* Hwp2p can complement the lack of filamentation of a *Saccharomyces cerevisiae flo11* null strain. Microbiology 159:1160–1164. doi:10.1099/mic.0.067249-0. 23558263

[34] Braun BR, Johnson AD 1997 Control of filament formation in *Candida albicans* by the transcriptional repressor TUP1. Science 277:105–109. doi:10.1126/science.277.5322.105. 9204892

[35] Braun BR, Head WS, Wang MX, Johnson AD 2000 Identification and characterization of TUP1-regulated genes in *Candida albicans*. Genetics 156:31–44. 10978273 10.1093/genetics/156.1.31 PMC1461230

[36] Legrand M, Chan CL, Jauert PA, Kirkpatrick DT 2007 Role of DNA mismatch repair and double-strand break repair in genome stability and antifungal drug resistance in *Candida albicans*. Eukaryot Cell 6:2194–2205. doi:10.1128/EC.00299-07. 17965250 PMC2168241

[37] García-Prieto F, Gómez-Raja J, Andaluz E, Calderone R, Larriba G 2010 Role of the homologous recombination genes RAD51 and RAD59 in the resistance of *Candida albicans* to UV light, radiomimetic and anti-tumor compounds and oxidizing agents. Fungal Genet Biol 47:433–445. doi:10.1016/j.fgb.2010.02.007. 20206282 PMC2852118

[38] Wang G, Tong X, Weng S, Zhou H 2012 Multiple phosphorylation of Rad9 by CDK is required for DNA damage checkpoint activation. Cell Cycle 11:3792–3800. doi:10.4161/cc.21987. 23070520 PMC3495822

[39] González-Prieto R, Muñoz-Cabello AM, Cabello-Lobato MJ, Prado F 2013 Rad51 replication fork recruitment is required for DNA damage tolerance. EMBO J 32:1307–1321. doi:10.1038/emboj.2013.73. 23563117 PMC3642682

[40] Alcasabas AA, Osborn AJ, Bachant J, Hu F, Werler PJH, Bousset K, Furuya K, Diffley JFX, Carr AM, Elledge SJ 2001 Mrc1 transduces signals of DNA replication stress to activate Rad53. Nat Cell Biol 3:958–965. doi:10.1038/ncb1101-958. 11715016

[41] Osborn AJ, Elledge SJ 2003 Mrc1 is a replication fork component whose phosphorylation in response to DNA replication stress activates Rad53. Genes Dev 17:1755–1767. doi:10.1101/gad.1098303. 12865299 PMC196183

[42] Andaluz E, Ciudad T, Gómez-Raja J, Calderone R, Larriba G 2006 Rad52 depletion in *Candida albicans* triggers both the DNA-damage checkpoint and filamentation accompanied by but independent of expression of hypha-specific genes. Mol Microbiol 59:1452–1472. doi:10.1111/j.1365-2958.2005.05038.x. 16468988

[43] Lockhart SR, Etienne KA, Vallabhaneni S, Farooqi J, Chowdhary A, Govender NP, Colombo AL, Calvo B, Cuomo CA, Desjardins CA, Berkow EL, Castanheira M, Magobo RE, Jabeen K, Asghar RJ, Meis JF, Jackson B, Chiller T, Litvintseva AP 2017 Simultaneous emergence of multidrug-resistant *Candida auris* on 3 continents confirmed by whole-genome sequencing and epidemiological analyses. Clin Infect Dis 64:134–140. doi:10.1093/cid/ciw691. 27988485 PMC5215215

[44] Chakrabarti A, Sood P, Rudramurthy SM, Chen S, Kaur H, Capoor M, Chhina D, Rao R, Eshwara VK, Xess I, Kindo AJ, Umabala P, Savio J, Patel A, Ray U, Mohan S, Iyer R, Chander J, Arora A, Sardana R, Roy I, Appalaraju B, Sharma A, Shetty A, Khanna N, Marak R, Biswas S, Das S, Harish BN, Joshi S, Mendiratta D 2015 Incidence, characteristics and outcome of ICU-acquired candidemia in India. Intensive Care Med 41:285–295. doi:10.1007/s00134-014-3603-2. 25510301

[45] Rhodes J, Abdolrasouli A, Farrer RA, Cuomo CA, Aanensen DM, Armstrong-James D, Fisher MC, Schelenz S 2018 Genomic epidemiology of the UK outbreak of the emerging human fungal pathogen *Candida auris*. Emerg Microbes Infect 43:1–12. doi:10.1038/s41426-018-0045-x. PMC5874254 29593275

[46] Zheng X, Wang Y, Wang Y 2004 Hgc1, a novel hypha-specific G1 cyclin-related protein regulates *Candida albicans* hyphal morphogenesis. EMBO J 23:1845–1856. doi:10.1038/sj.emboj.7600195. 15071502 PMC394249

[47] Braun BR, Kadosh D, Johnson AD 2001 NRG1, a repressor of filamentous growth in *C. albicans*, is down-regulated during filament induction. EMBO J 20:4753–4761. doi:10.1093/emboj/20.17.4753. 11532939 PMC125265

[48] Kim SH, Iyer KR, Pardeshi L, Muñoz JF, Robbins N, Cuomo CA, Wong KH, Cowen LE 2019 Genetic analysis of *Candida auris* implicates Hsp90 in morphogenesis and azole tolerance and Cdr1 in azole resistance. mBio 10:e02529-18. doi:10.1128/mBio.02529-18. 30696744 PMC6355988

[49] Bachewich C, Thomas DY, Whiteway M 2003 Depletion of a Polo-like kinase in *Candida albicans* activates Cyclase-dependent hyphal-like growth. Mol Biol Cell 14:2163–2180. doi:10.1091/mbc.02-05-0076. 12802083 PMC165105

[50] Chen C, Zeng G, Wang Y 2018 G1 and S phase arrest in *Candida albicans* induces filamentous growth via distinct mechanisms. Mol Microbiol 110:191–203. doi:10.1111/mmi.14097. 30084240

[51] Bacal J, Moriel‐Carretero M, Pardo B, Barthe A, Sharma S, Chabes A, Lengronne A, Pasero P 2018 Mrc1 and Rad9 cooperate to regulate initiation and elongation of DNA replication in response to DNA damage. EMBO J 37:1–18. doi:10.15252/embj.201899319. 30158111 PMC6213276

[52] Lopes M, Cotta-Ramusino C, Liberi G, Foiani M 2003 Branch migrating sister chromatid junctions form at replication origins through Rad51/Rad52-independent mechanisms. Mol Cell 12:1499–1510. doi:10.1016/s1097-2765(03)00473-8. 14690603

[53] Zou L, Elledge SJ 2003 Sensing DNA damage through ATRIP recognition of RPA-ssDNA complexes. Science 300:1542–1548. doi:10.1126/science.1083430. 12791985

[54] Hashimoto Y, Chaudhuri AR, Lopes M, Costanzo V 2010 Rad51 protects nascent DNA from Mre11-dependent degradation and promotes continuous DNA synthesis. Nat Struct Mol Biol 17:1305–1311. doi:10.1038/nsmb.1927. 20935632 PMC4306207

[55] Petermann E, Orta ML, Issaeva N, Schultz N, Helleday T 2010 Hydroxyurea-stalled replication forks become progressively inactivated and require two different RAD51-mediated pathways for restart and repair. Mol Cell 37:492–502. doi:10.1016/j.molcel.2010.01.021. 20188668 PMC2958316

[56] Prado F 2018 Homologous recombination: to fork and beyond. Genes (Basel) 9:603. doi:10.3390/genes9120603. PMC6316604 30518053

[57] Zaragoza O, Nielsen K 2013 Titan cells in *Cryptococcus neoformans*: cells with a giant impact. Curr Opin Microbiol 16:409–413. doi:10.1016/j.mib.2013.03.006. 23588027 PMC3723695

[58] Dambuza IM, Drake T, Chapuis A, Zhou X, Correia J, Taylor-Smith L, LeGrave N, Rasmussen T, Fisher MC, Bicanic T, Harrison TS, Jaspars M, May RC, Brown GD, Yuecel R, MacCallum DM, Ballou ER 2018 The *Cryptococcus neoformans* Titan cell is an inducible and regulated morphotype underlying pathogenesis. PLoS Pathog 14:e1006978. doi:10.1371/journal.ppat.1006978. 29775474 PMC5959070

[59] Malavia D, Lehtovirta-Morley LE, Alamir O, Weiß E, Gow NAR, Hube B, Wilson D 2017 Zinc limitation induces a hyper-adherent Goliath phenotype in *Candida albicans*. Front Microbiol 8:2238. doi:10.3389/fmicb.2017.02238. 29184547 PMC5694484

[60] Loll-Krippleber R, Enfert C, Feri A, Diogo D, Perin A, Marcet-Houben M, Bougnoux M, Legrand M 2014 A study of the DNA damage checkpoint in *Candida albicans*: uncoupling of the functions of Rad53 in DNA repair, cell cycle regulation and genotoxic stress-induced polarized growth. Mol Microbiol 91:452–471. doi:10.1111/mmi.12471. 24286230

[61] Legrand M, Chan CL, Jauert PA, Kirkpatrick DT 2011 The contribution of the S-phase checkpoint genes MEC1 and SGS1 to genome stability maintenance in *Candida albicans*. Fungal Genet Biol 48:823–830. doi:10.1016/j.fgb.2011.04.005. 21511048 PMC3126902

[62] Katou Y, Kanoh Y, Bando M, Noguchi H, Tanaka H, Ashikari T, Sugimoto K, Shirahige K 2003 S-phase checkpoint proteins Tof1 and Mrc1 form a stable replication-pausing complex. Nature 424:1078–1083. doi:10.1038/nature01900. 12944972

[63] Masai H, Yang CC, Matsumoto S 2017 Mrc1/Claspin: a new role for regulation of origin firing. Curr Genet 63:813–818. doi:10.1007/s00294-017-0690-y. 28357499

[64] Miller CT, Gabrielse C, Chen YC, Weinreich M 2009 Cdc7p-Dbf4p regulates mitotic exit by inhibiting Polo kinase. PLoS Genet 5:e1000498. doi:10.1371/journal.pgen.1000498. 19478884 PMC2682205

[65] Asano S, Park JE, Sakchaisri K, Yu LR, Song S, Supavilai P, Veenstra TD, Lee KS 2005 Concerted mechanism of Swe1/Wee1 regulation by multiple kinases in budding yeast. EMBO J 24:2194–2204. doi:10.1038/sj.emboj.7600683. 15920482 PMC1150880

[66] Zhang T, Nirantar S, Lim HH, Sinha I, Surana U 2009 DNA damage checkpoint maintains Cdh1 in an active state to inhibit anaphase progression. Dev Cell 17:541–551. doi:10.1016/j.devcel.2009.09.006. 19853567

[67] Simpson-Lavy KJ, Brandeis M 2010 Clb2 and the APC/CCdh1 regulate Swe1 stability. Cell Cycle 9:3046–3053. doi:10.4161/cc.9.115.12457. 20714223

[68] Pruyne D, Bretscher A 2000 Polarization of cell growth in yeast. I. Establishment and maintenance of polarity states. J Cell Sci 113:365–375. 10639324 10.1242/jcs.113.3.365

[69] Lew DJ 2003 The morphogenesis checkpoint: how yeast cells watch their figures. Curr Opin Cell Biol 15:648–653. doi:10.1016/j.ceb.2003.09.001. 14644188

[70] Ahn SH, Acurio A, Kron SJ 1999 Regulation of G2/M progression by the STE mitogen-activated protein kinase pathway in budding yeast filamentous growth. Mol Biol Cell 10:3301–3316. doi:10.1091/mbc.10.10.3301. 10512868 PMC25595

[71] Bensen ES, Clemente-Blanco A, Finley KR, Correa-Bordes J, Berman J 2005 The mitotic cyclins Clb2p and Clb4p affect morphogenesis in Candida albicans. Mol Biol Cell 16:3387–3400. doi:10.1091/mbc.e04-12-1081. 15888543 PMC1165420

[72] Wu X, Jiang YW 2005 Possible integration of upstream signals at Cdc42 in filamentous differentiation of *S. cerevisiae*. Yeast 22:1069–1077. doi:10.1002/yea.1294. 16200521

[73] Shapiro RS, Cowen LE 2010 Coupling temperature sensing and development: hsp90 regulates morphogenetic signaling in *Candida albicans*. Virulence 1:45–48. doi:10.4161/viru.1.1.10320. 21178413 PMC3080193

[74] Khurana N, Laskar S, Bhattacharyya MK, Bhattacharyya S 2016 Hsp90 induces increased genomic instability toward DNA-damaging agents by tuning down RAD53 transcription. Mol Biol Cell 27:2463–2478. doi:10.1091/mbc.E15-12-0867. 27307581 PMC4966986

[75] Bravo Ruiz G, Ross ZK, Holmes E, Schelenz S, Gow NAR, Lorenz A 2019 Rapid and extensive karyotype diversification in haploid clinical *Candida auris* isolates. Curr Genet 65:1217–1228. doi:10.1007/s00294-019-00976-w. 31020384 PMC6744574

[76] Lee KL, Buckley HR, Campbell CC 1975 An amino acid liquid synthetic medium for the development of mycellal and yeast forms of *Candida albicans*. Sabouraudia 13:148–153. doi:10.1080/00362177585190271. 808868

[77] Fortuna M, Sousa MJ, Corte-Real M, Leao C, Salvador A, Sansonetty F 2000 Cell cycle analysis of yeasts. Curr Protoc Cytom Chapter 11:Unit 11.13. doi:10.1002/0471142956.cy1113s13. 18770687

[78] Burke D, Dawson D, Stearns T 2000 Methods in yeast genetics. Cold Spring Harbor Laboratory Press, Cold Spring Harbor, NY.

[79] Vyas VK, Barrasa MI, Fink GR 2015 A *Candida albicans* CRISPR system permits genetic engineering of essential genes and gene families. Sci Adv 1:e1500248. doi:10.1126/sciadv.1500248. 25977940 PMC4428347

[80] Shevchuk NA, Bryksin AV, Nusinovich YA, Cabello FC, Sutherland M, Ladisch S 2004 Construction of long DNA molecules using long PCR-based fusion of several fragments simultaneously. Nucleic Acids Res 32:e19. doi:10.1093/nar/gnh014. 14739232 PMC373371

[81] Walther A, Wendland J 2003 An improved transformation protocol for the human fungal pathogen *Candida albicans*. Curr Genet 42:339–343. doi:10.1007/s00294-002-0349-0. 12612807

[82] Chatterjee S, Alampalli SV, Nageshan RK, Chettiar ST, Joshi S, Tatu US 2015 Draft genome of a commonly misdiagnosed multidrug resistant pathogen *Candida auris*. BMC Genomics 16:686. doi:10.1186/s12864-015-1863-z. 26346253 PMC4562351

[83] Waterhouse AM, Procter JB, Martin DMA, Clamp M, Barton GJ 2009 Jalview version 2—a multiple sequence alignment editor and analysis workbench. Bioinformatics 25:1189–1191. doi:10.1093/bioinformatics/btp033. 19151095 PMC2672624

[84] Li H, Durbin R 2010 Fast and accurate long-read alignment with Burrows-Wheeler transform. Bioinformatics 26:589–595. doi:10.1093/bioinformatics/btp698. 20080505 PMC2828108

[85] Li H, Handsaker B, Wysoker A, Fennell T, Ruan J, Homer N, Marth G, Abecasis G, Durbin R, 1000 Genome Project Data Processing Subgroup. 2009 The Sequence Alignment/Map format and SAMtools. Bioinformatics 25:2078–2079. doi:10.1093/bioinformatics/btp352. 19505943 PMC2723002

[86] Walker BJ, Abeel T, Shea T, Priest M, Abouelliel A, Sakthikumar S, Cuomo CA, Zeng Q, Wortman J, Young SK, Earl AM 2014 Pilon: an integrated tool for comprehensive microbial variant detection and genome assembly improvement. PLoS One 9:e112963. doi:10.1371/journal.pone.0112963. 25409509 PMC4237348

[87] Stanke M, Steinkamp R, Waack S, Morgenstern B 2004 AUGUSTUS: a web server for gene finding in eukaryotes. Nucleic Acids Res 32:W309–W312. doi:10.1093/nar/gkh379. 15215400 PMC441517

